# The effect of distal tibial tuberosity high tibial osteotomy on postoperative patellar height and patellofemoral joint degeneration

**DOI:** 10.1186/s13018-020-01996-w

**Published:** 2020-10-09

**Authors:** Changxiao Han, Xia Li, Xiangdong Tian, Jiping Zhao, Liqun Zhou, Yetong Tan, Sheng Ma, Yuanyi Hu, Handong Chen, Ye Huang

**Affiliations:** 1 Beijing University of Chinese Medicine, Beijing, 100029 China; 2 Academe of Wudang Medicine of Beijing University of Chinese Medicine, Beijing, 100029 China; 3 Minimal Invasive Joint Department, Beijing University of Chinese Medicine Third Affiliated Hospital, No. 51 Anwai Xiaoguan Street, Chaoyang District, Beijing, 100029 China; 4 Beijing University of Chinese Medicine Affiliated Dongzhimen Hospital, Beijing, 100070 China

**Keywords:** Patella height, Patellofemoral joint, High tibial osteotomy, Distal tibial tuberosity, Medial compartmental knee osteoarthritis

## Abstract

**Background:**

Distal tibial tuberosity high tibial osteotomy (DTT-HTO) can prevent distalization of the tibial tuberosity and thus patellar infera. However, no studies on the clinical and radiological effects of DTT-HTO on the patellofemoral joint have been conducted. The purpose of the study was to evaluate the effect of DTT-HTO on patella height and patellofemoral joint congruity based on the severity of patellofemoral joint OA.

**Methods:**

Twenty-nine patients (33 knees) who underwent DTT-HTO and second-look arthroscopy when implant was removed between January 2018 and May 2020 were eligible for the study. Among them, 6 were males, and 23 were females, with ages from 51 to 78 years old. The Caton-Deschamps index (CDI), congruence angle (CA), and lateral patellar tilt (LPT) were measured to evaluate the effect of surgery on patellar height and patellofemoral joint congruity. The weight-bearing line ratio (WBLR) was measured to assess lower limb alignment. The cartilage lesion in the patellofemoral joint was assessed arthroscopically during surgery and implant removal by the International Cartilage Repair Society (ICRS) grading system at 18–24 months after surgery. The Hospital for Special Surgery (HSS) scale was used to evaluate knee joint function.

**Results:**

Twenty-nine patients were followed up for 18–28 months. The preoperative CDI, CA, and LPT changed from 0.92 ± 0.16 to 0.89 ± 0.14, from 5.52 ± 2.19 to 5.44 ± 2.27, and from 6.95 ± 2.88 to 6.54 ± 2.42, respectively, and the differences were not statistically significant (*p* > 0.05). The preoperative WBLR significantly increased from 16.72 ± 6.77 to 58.77 ± 7.69% (*p* < 0.001). The cartilage lesions in the patella and femoral trochlea did not progress significantly from the first- to the second-look arthroscopy, according to the ICRS grades (*p* > 0.05). The HSS score significantly improved from 50.64 ± 19.18 preoperatively to 67.33 ± 14.72, 81.63 ± 11.92, and 82.73 ± 8.05 at the 3-month, 12-month, and last follow-up after surgery (*p* < 0.001).

**Conclusion:**

DTT-HTO can effectively prevent patellar infera, and its effects on postoperative patellofemoral joint congruity and patellofemoral joint OA progression are inconspicuous. It can be recommended as a treatment of varus knee combined with patellar infera or patellofemoral joint OA.

## Background

Open-wedge high tibial osteotomy (OWHTO) is an effective treatment for medial compartment knee osteoarthritis and has been reported to yield good long-term clinical outcomes [1]. OWHTO can cure genu varum by adjusting the weight-bearing line, thereby transferring the pressure from the medial compartment to the lateral compartment. Therefore, the significant change in surgery is alteration of the alignment on the coronal plane. In addition, the patellofemoral joint on the sagittal plane is also influenced by the operation [2]. Several studies have shown that OWHTO causes patellar infera, increases patellofemoral joint pressure pain, increases the risk of patellofemoral OA progression, and makes it more difficult for total knee arthroplasty to be performed in the future [3–8]. Kim et al. [8] reported the prevalence of the progression of patellofemoral OA to be 41.2% in the femoral trochlea and 21.9% in the patella articular surface after more than 2 years in 114 patients who underwent OWHTO.

Patellar infera and patellofemoral malalignment have been reported to result from tibial tuberosity distalization and patellar tendon adherence [7, 9]. To prevent patella infera, several studies have reported that osteotomy in the distal tibial tuberosity or below the tuberosity should be performed [10–13]. However, the osteotomy region involves a wide area of cortical bone, so fixation is also needed to provide a sufficient anti-rotational force. In addition, osteotomy in cortical bone may decrease the rate of bone healing. In recent years, with the development of new medical instruments, a new patented π-plate [14] has been invented for additional fixation in distal tibial tuberosity high tibial osteotomy (DTT-HTO).

DTT-HTO performed with the π-plate has good clinical efficacy and can be used in a broad range of applications since the surgery has little effect on the patella, leading to a greater range of correction; moreover, the operation is simple to perform. However, to our knowledge, no studies on patellar height and patellofemoral joint congruity after DTT-HTO performed with π-plates have been conducted to date. Therefore, the present study aimed to determine the clinical and radiographic effects of DTT-HTO on postoperative patellar height and patellofemoral joint congruity with respect to the severity of patellofemoral joint OA.

## Methods

### Patients

Patients who underwent DTT-HTO and second-look arthroscopy when implant was removed for the treatment of medial compartment knee osteoarthritis with a varus deformity between January 2018 and May 2020 were eligible for this retrospective study. The inclusion criteria included surgical indications for HTO: (1) a Kellgren-Lawrence grade [15] for medial compartment knee osteoarthritis ≥ III, (2) a lateral compartment cartilage that is relatively intact with a Kellgren-Lawrence grade ≤ I, (3) a varus deformity ≥ 5°, (4) a patella without severe deviations in positioning with a patellofemoral joint Kellgren-Lawrence grade ≤ III, and (5) complete postoperative follow-up and radiological data. The exclusion criteria were as follows: (1) a severe ligament injury of the affected knee joint, (2) a history of arthroscopy before DTT-HTO for lateral retinacular release, (3) a history of surgery on the affected knee before DTT-HTO, (4) severe osteoporosis or rheumatoid arthritis, and (5) secondary injuries of the affected knee during the follow-up period. Finally, 29 patients and 33 knees (from 6 men and 23 women) were enrolled in this study. The mean patient age was 61.7 ± 6.2 years (range, 54–76) (Table 1).

**Table 1:** Demographics of the patients

Demographics	
Number of patients (*n*)	29 (33knees)
Mean age (years)	64.25 ± 7.19
Male/female	6/23
Left/right	18/15
Duration of follow-up (months)	23.42 ± 4.71
Height (cm)	161.79 ± 6.82
Weight (kg)	64.91 ± 8.22
BMI (kg/m^2^)	26.17 ± 4.38
Disease course (years)	4.28 ± 2.14
ROM (°)	127.43 ± 26.62
Varus deformity (°)	9.28 ± 4.14

### Surgical techniques

**Fig. 1:**
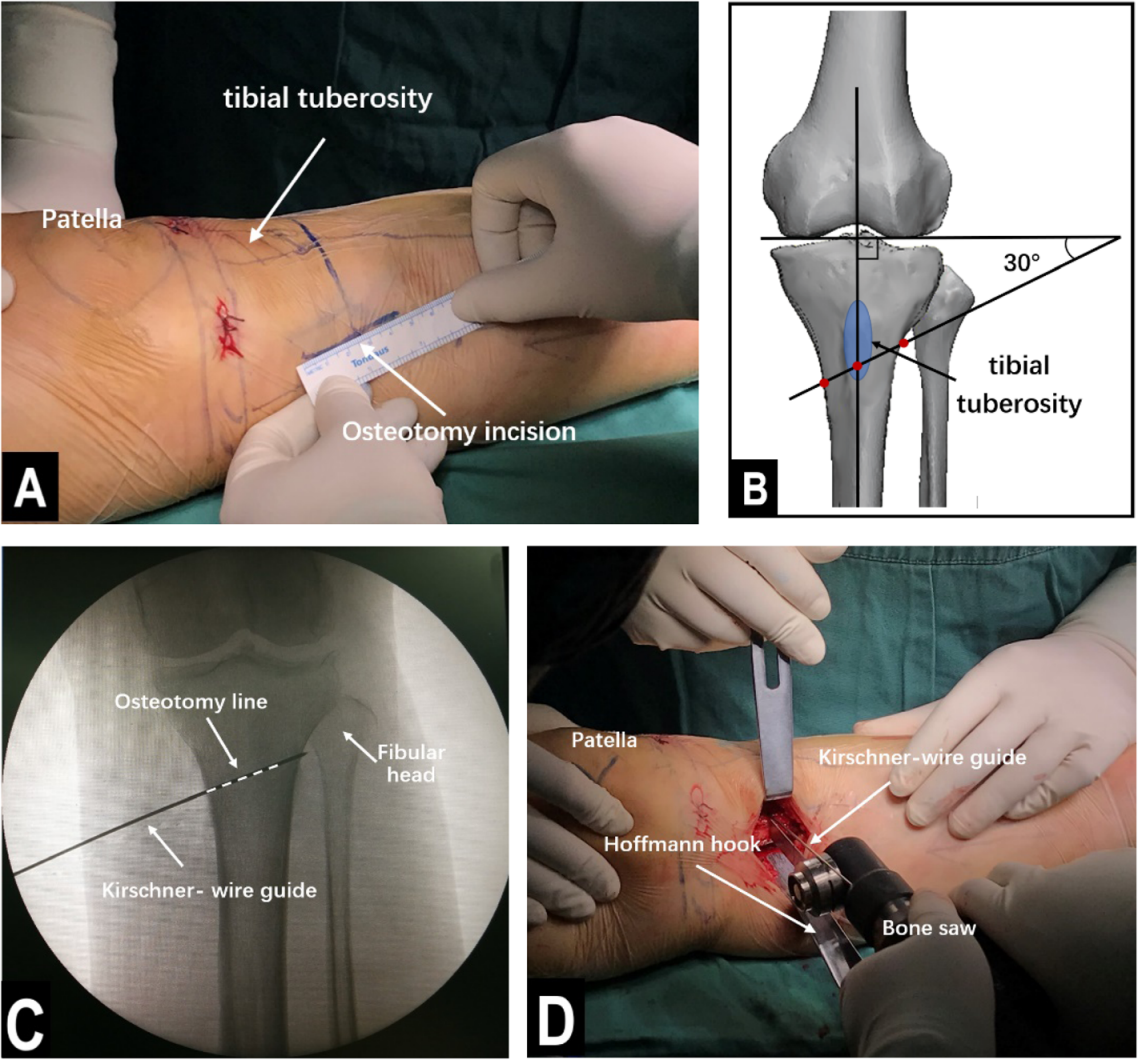
Location of the osteotomy region and osteotomy. **a** The incision, which was approximately 5 cm long and made according to the surface landmark. **b** A 3D model of the bone showing where the osteotomy line was located. **c** C-arm image after the osteotomy line was located by the Kirschner wire guide. **d** A bone saw, which was used to perform the osteotomy guided by the Kirschner wire guide

**Fig. 2:**
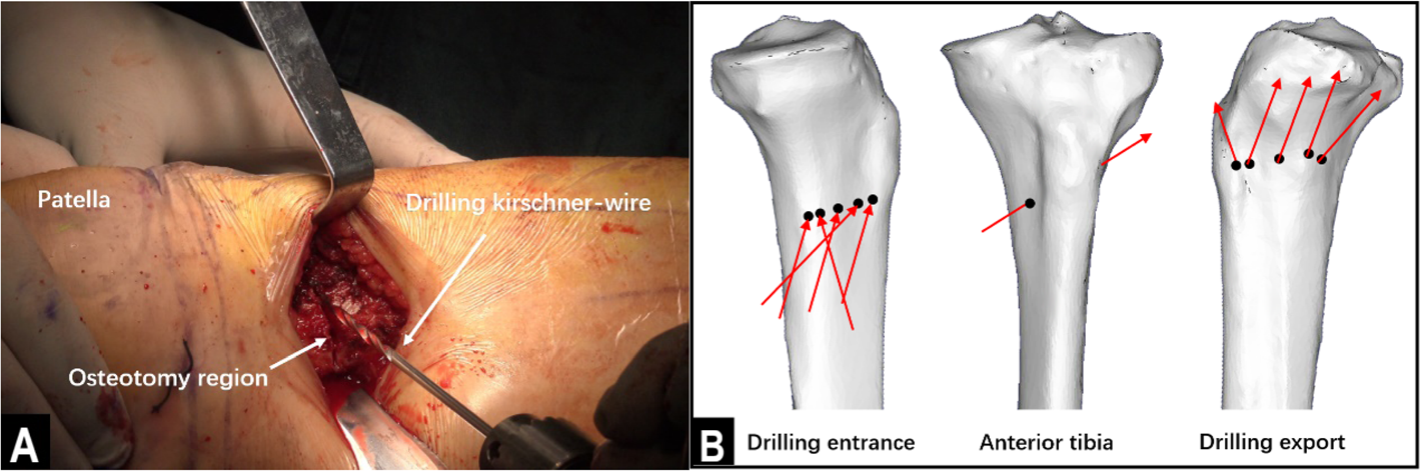
Lessening the stress on the bone cortex. **a** A Kirschner wire approximately 2.8 mm wide was used to drill 5 holes on the lateral bone cortex. **b** Three 3D models of the bone showing how the holes along the transverse plane were drilled

**Fig. 3:**
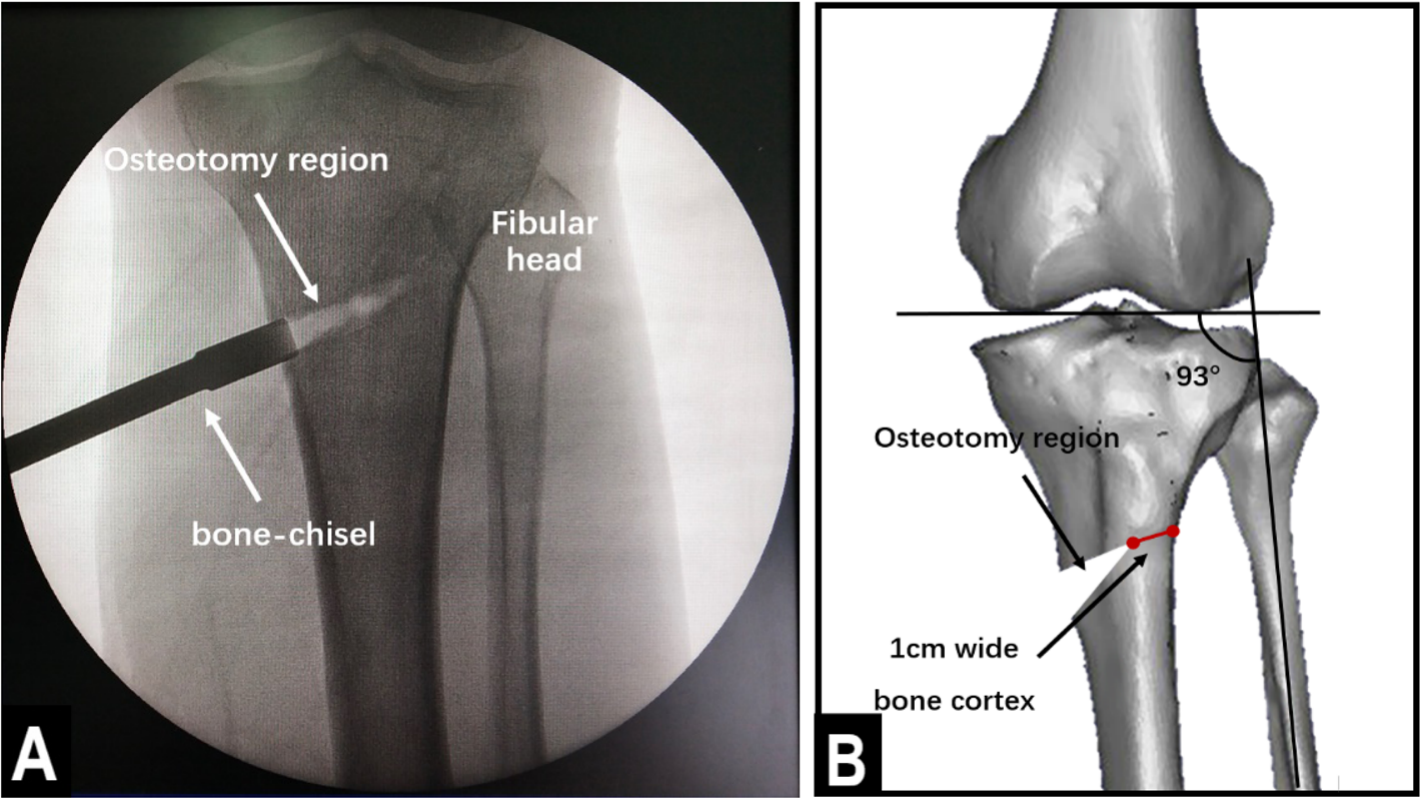
Opening the osteotomy region. **a** C-arm image after the osteotomy region was opened. **b** A 3D model of the bone showing the opening in detail. The width of the lateral part of the intact bone cortex was maintained to be 1 cm, while the intersecting angle between the femoral condyles and the fibula axis was adjusted to 93°

**Fig. 4:**
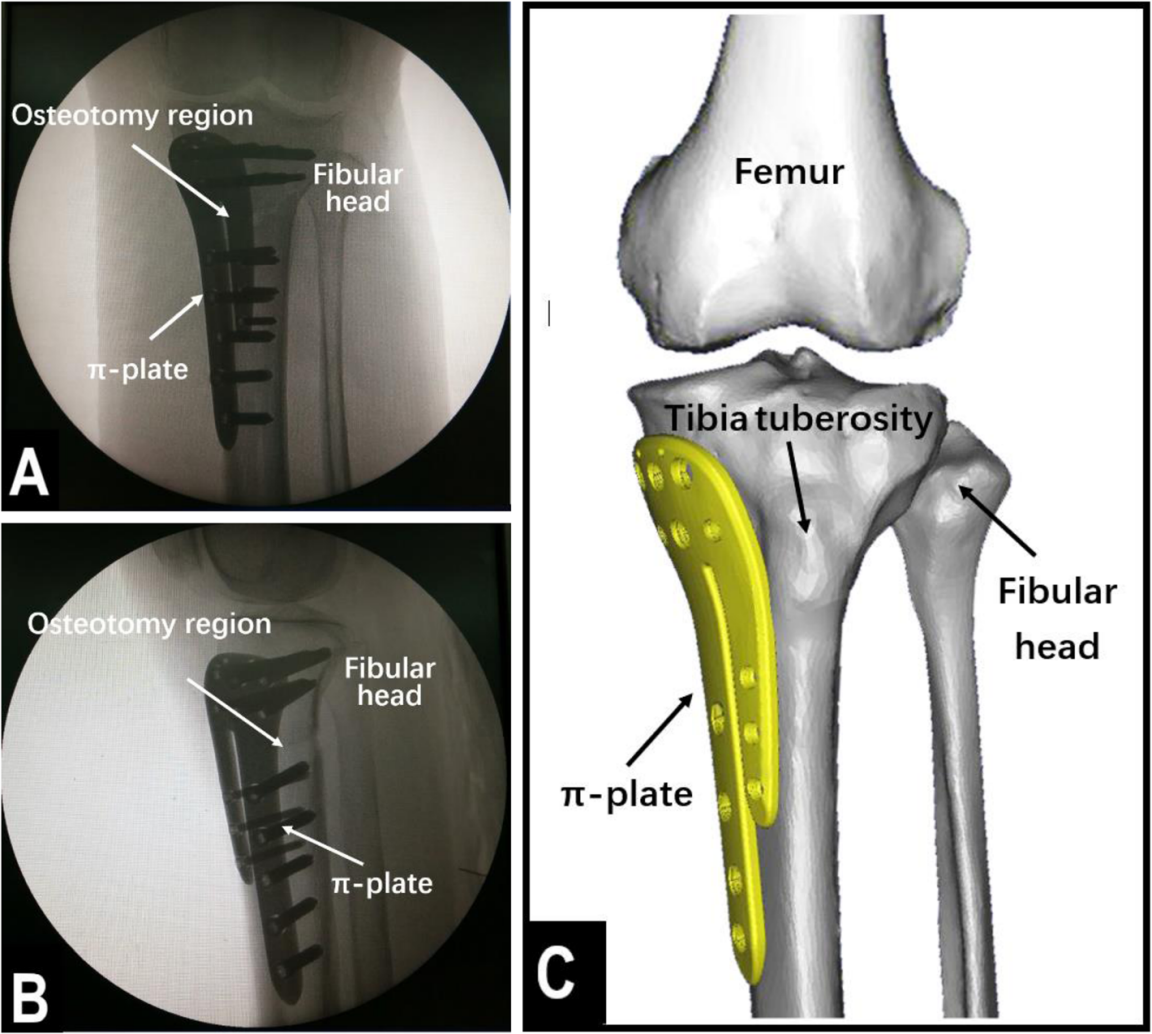
Using the π-plate to secure the osteotomy region. **a** C-arm anteroposterior image after the osteotomy region was locked. **b** C-arm lateral image after the osteotomy region was locked. **c** A 3D model of the bone, showing the position of the π-plate

The operations were performed by the same group of experienced physicians. The patient was placed in a supine positioned, and subarachnoid block anesthesia combined with continuous epidural anesthesia was used. Before the osteotomy, an arthroscopic examination was performed to evaluate the cartilage of the medial and lateral compartments and the patellofemoral joint. Arthroscopic debridement, including meniscectomy or synovectomy, was performed if necessary. Then, distal tibial tuberosity high tibial osteotomy was performed. First, a longitudinal incision approximately 5 cm long was made at 1 cm below the anterior medial knee joint space, in accordance with the osteotomy position determined by the surface landmark. The incisions were made layer by layer to reveal the pes anserinus, the medial collateral ligament, the periosteum, and the tibia. Then, a Kirschner wire guide was inserted along the exposed proximal tibia toward the tibial fibular fornix. Under c-arm fluoroscopy, the Kirschner wire was passed through the distal tibial tubercle to approximately 0.5 cm below the tibial fibular fornix. The angle between the Kirschner wire guide and the line connecting the tips of the two femoral condyles (horizontal line of the tibial plateau) was 30°. The osteotomy line was made in the direction of the Kirschner wire guide. A Hoffmann hook was placed in the lateral tibia along the osteotomy line to protect the lateral vasculature and nerves. Then, a bone saw was used to perform the osteotomy along the direction of the osteotomy line, as determined by the Kirschner wire guide, and the outermost 1 cm of the lateral side of the tibia was left intact (Fig. 1). In the lateral part of the intact tibia, 5 holes were drilled with 2.8-mm Kirschner wires to lessen the stress on the lateral cortical bone, thus preventing the bone from fracturing during the surgical procedure (Fig. 2). An assistant was positioned on the opposite side of the surgeon, with one hand placed against the lateral part of the intact tibia and the other hand holding the ankle to help the surgeon open the osteotomy region. Meanwhile, the soles of the feet were turned outwards to relax the tibialis anterior muscle. Under c-arm fluoroscopy, the intersecting angle between the femoral condyles and the fibula axis was adjusted to 93° (Fig. 3). Finally, fixation of the osteotomy was conducted with the π-plate and locking screws (Fig. 4).

For postoperative rehabilitation, active ankle exercises were initiated immediately after DTT-HTO, and knee flexion-extension exercises and straight-leg raise exercises were commenced 1 day after surgery. Toe-touch weight bearing was initiated 2 days after the surgery. The patients were allowed to gradually start partial weight-bearing exercises at 1–4 weeks, and full weight-bearing exercises at 6–8 weeks postoperatively. In addition, implant removal was planned for 18–24 months after surgery, and arthroscopy was also performed before implant removal to determine the status of the cartilage.

### Radiological evaluation

**Fig. 5:**
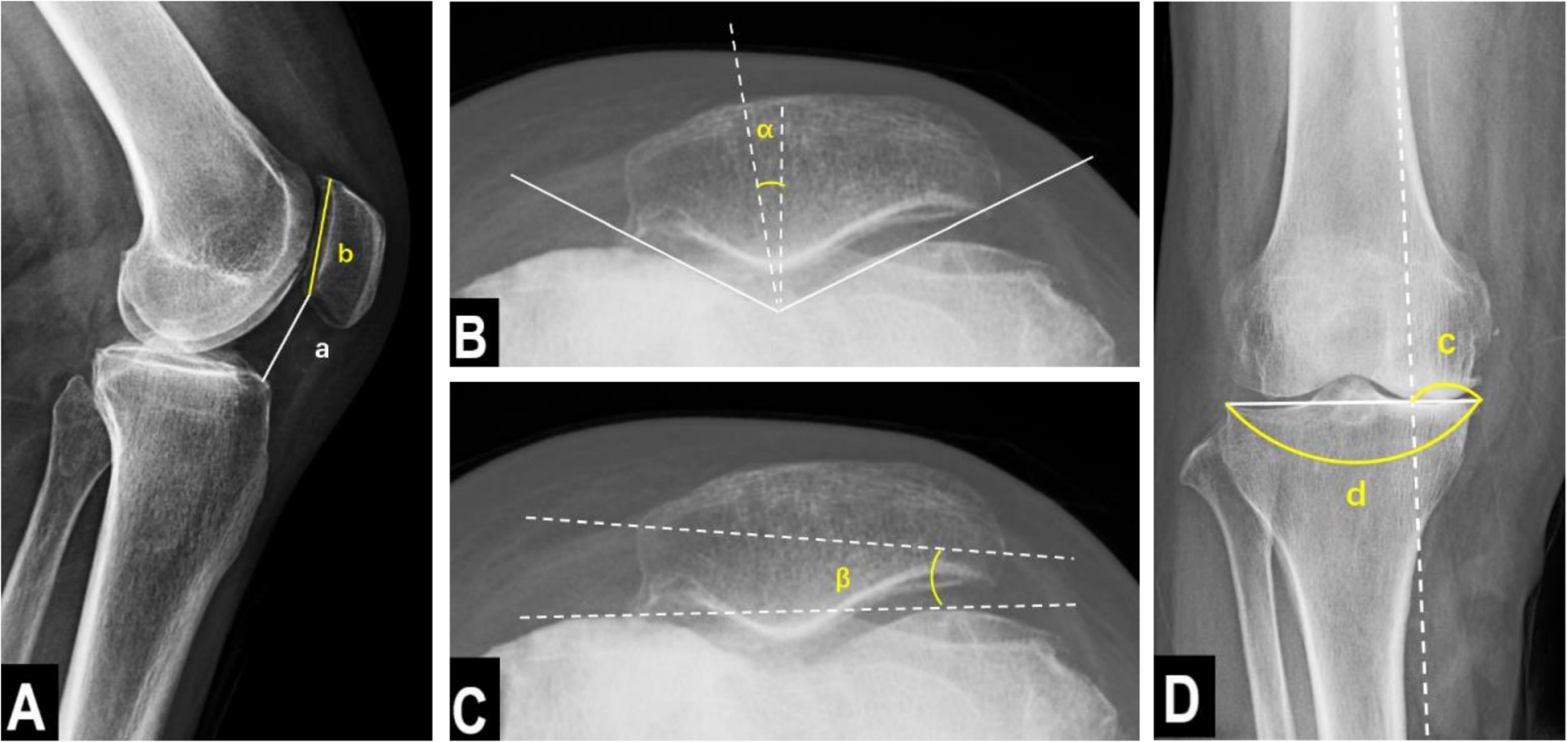
Radiological measurements of knee joint. **A** The Caton-Deschamps index (CDI) = a/b. CDI is the length from the distal end of the patellar joint surface to the anterior tip of the tibial tuberosity, divided by the length of the patellar joint surface. **B** Congruence angle (CA) = α. CA is the angle between the line bisecting the sulcus angle and the line that links the lowest point of the intercondylar sulcus to the lowest point on the articular ridge of the patella. **C** Lateral patellar tilt (LPT) = β. LPT is the angle between the line intersecting the widest bony structure of the patella and the line tangentially passing the anterior surface of the femoral condyles. **D** The weight-bearing line ratio (WBLR) = c/d. WBLR is the ratio of the horizontal distance from the medial edge of the tibial plateau to the intersection of the weight-bearing line and the entire length of the proximal tibial plateau line

A radiological evaluation was performed preoperatively and at the last follow-up after surgery. All the radiological data were recorded by a dedicated radiology technician under the supervision of 2 orthopedic surgeons who did not participate in the surgery. The Caton-Deschamps index (CDI: the length from the distal end of the patellar joint surface to the anterior tip of the tibial tuberosity divided by the length of the patellar joint surface) was measured using a lateral view of the knee to evaluate the patellar height. The congruence angle (CA: the angle between the zero-reference line bisecting the sulcus angle and the line that links the lowest point of the intercondylar sulcus to the lowest point on the articular ridge of the patella) and lateral patellar tilt (LPT: the angle between the line intersecting the widest bony structure of the patella and the line crossing the anterior surfaces of the femoral condyles tangentially) were measured to evaluate the patellofemoral joint congruity in a skyline view of the knee. Full-length lower extremity weight-bearing radiographs were obtained to determine the weight-bearing line ratio (WBLR: the horizontal distance from the WBL to the medial edge of the tibial plateau, divided by the width of the tibial tibia, with the ratio at the medial tibial edge being 0% and that at the lateral tibial edge being 100%) Fig. 5.

### Evaluation of the cartilage in the patellofemoral joint

The statuses of the chondral lesions in the patella and femoral trochlea were recorded through arthroscopy at the initial operation and implant removal, and the arthroscopic images were stored for reference. The status of the articular cartilage was evaluated by the International Cartilage Repair Society (ICRS: grade 0, normal; grade 1, softening of the articular cartilage and superficial lacerations and fissures; grade 2, fragmentation and fissuring that extends < 50% of the articular cartilage thickness; grade 3, fragmentation and fissuring that extends > 50% of the articular cartilage thickness; grade 4, complete loss of articular cartilage thickness).

### Clinical evaluation

The Hospital for Special Surgery (HSS) scale was used to evaluate the clinical outcomes preoperatively and at the 3-month, 12-month, and last follow-up after surgery. The items of the scale pertain to pain, function, activity, muscle strength, flexion deformity, and stability. The maximum score for the HSS is 100 points.

### Statistical analysis

SPSS (version 20.0, IBM China) was used for statistical analysis. The comparisons of CDI, CA, PTA, and WBLR preoperatively and at the last follow-up after surgery were performed by the paired-samples *T* test. Repeated measures ANOVA was used to analyze the changes in the HSS score preoperatively and at the 3-month, 12-month, and last follow-up after surgery. To compare the ICRS grades of the patellofemoral articular cartilage based on the arthroscopic findings, the chi-square test or Fisher’s exact test was used. *p* < 0.05 was considered statistically significant.

## Results

The CDI changed from 0.92 ± 0.16 preoperatively to 0.89 ± 0.14 at the last follow-up. The preoperative and postoperative CDI values were not significantly different (*p* > 0.05). The CA and LPT changed from 5.52 ± 2.19 to 5.44 ± 2.27 and from 6.95 ± 2.88 to 6.54 ± 2.42, respectively. The CA and LPT values did not significantly change from pre- to postoperatively (*p* > 0.05). The preoperative WBLR significantly changed from 16.72 ± 6.77 to 58.77 ± 7.69% (*p* < 0.001). The average degree of correction was 42%, as assessed by the change in the WBLR (Table 2).

**Table 2:** Radiographic evaluations performed before and after distal tibial tuberosity high tibial osteotomy

	Preoperative	Last follow-up	Value	*p* value
CDI	0.92 ± 0.16	0.89 ± 0.14	*t* = 0.379	0.314
CA	5.52 ± 2.19	5.44 ± 2.27	*t* = 0.529	0.276
LPT	6.95 ± 2.88	6.54 ± 2.42	*t* = 0.873	0.441
WBLR	16.72 ± 6.77	58.77 ± 7.69	*t* = 2.741	< 0.001

**Fig. 6:**
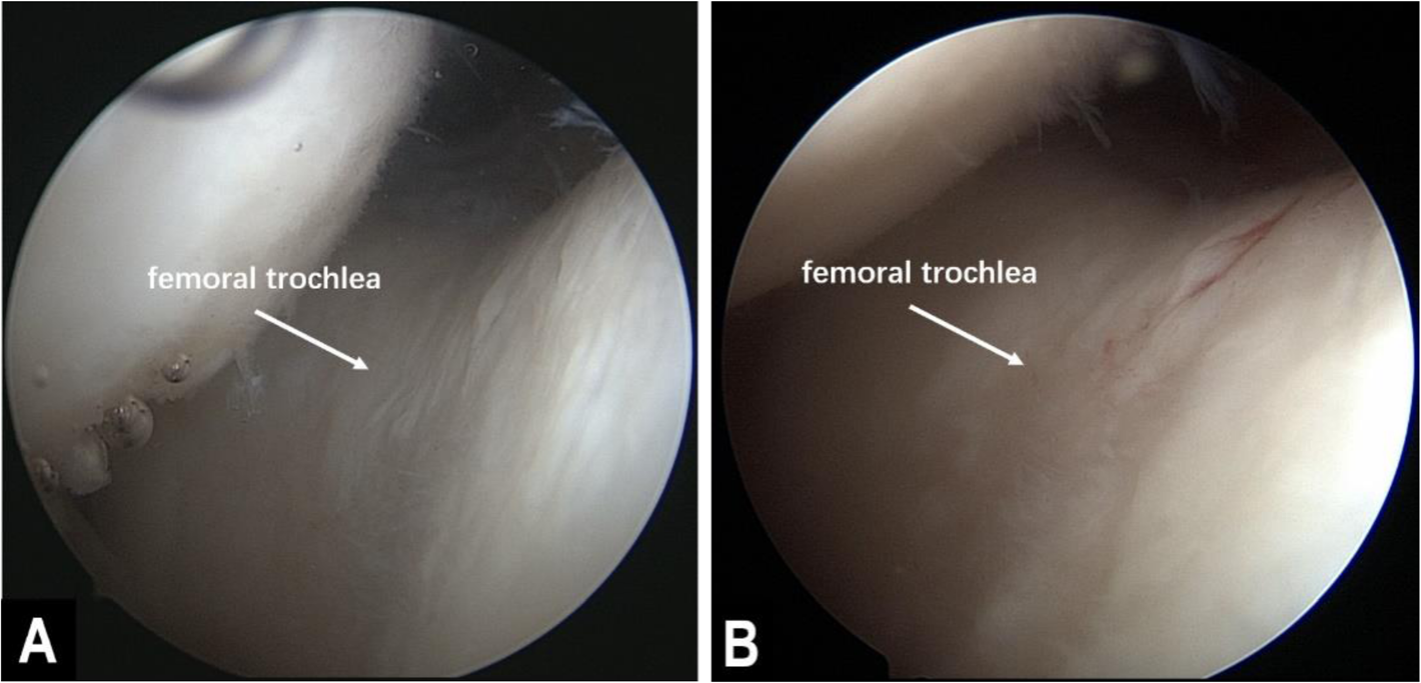
Images of two arthroscopy procedures performed in the right knee of a 57-year-old woman. **a** The first arthroscopy performed during the osteotomy showed cartilage lesions with an ICRS grade of 2 on the femoral trochlea. **b** The second-look arthroscopy was performed during implant removal at 25 months postoperatively and showed that there was no significant degeneration in the femoral trochlear cartilage

The cartilage lesions in the patella and femoral trochlea did not significantly progress from the first- to the second-look arthroscopy. For the ICRS grade of the patella, 3, 11, 9, and 6 cases during the first arthroscopy and 1, 12, 8, and 8 cases during the second arthroscopy had grades of 0, 1, 2, and 3, respectively (*p* > 0.05). For the ICRS grade of the femoral trochlea, 2, 15, 7, and 5 cases during the first arthroscopy and 1, 15, 8, and 5 cases during the second arthroscopy had grades of 0, 1, 2, and 3, respectively (*p* > 0.05). The HSS score significantly improved from 50.64 ± 19.18 preoperatively to 67.33 ± 14.72, 81.63 ± 11.92, and 82.73 ± 8.05 at the 3-month, 12-month, and last follow-up after surgery (*p* < 0.001) (Table 3, Fig. 6).

**Table 3:** Clinical evaluation of the HSS score and progression of patellofemoral articular cartilage lesions, as measured by the ICRS grade

		Value	*p* value
ICRS grade in the femoral trochlea (0/1/2/3)			
1st look	3/11/9/6		
2nd look	1/12/8/8	*χ*^2^ = 2.766	0.449
ICRS grade in the patella (0/1/2/3)			
1st look	2/15/7/5		
2nd look	1/15/8/5	*χ*^2^ = 1.495	0.318
HSS score			
Preoperatively	50.64 ± 19.18		
3 months postoperatively	67.33 ± 14.72		
12 months postoperatively	81.63 ± 11.92		
Last follow-up	82.73 ± 8.05	*F* = 221.742	< 0.001

## Discussion

The present study focused on the effect of distal tibial tuberosity high tibial osteotomy (DTT-HTO) on patellar height and patellofemoral joint congruity with respect to the severity of patellofemoral joint OA. The key finding in this study was that DTT-HTO did not significantly change the patellar height or patellofemoral joint congruity, and the cartilage lesions of the patellofemoral joint did not tend to deteriorate. In addition, the weight-bearing line of the patients was effectively adjusted after the surgery, yielding good clinical outcomes.

Patellar infera and patellofemoral joint OA progression are frequently occurring complications of OWHTO. Tibial tuberosity distalization and patellar tendon adherence, which cause patellar infera, have been well demonstrated anatomically and biomechanically [9, 16]. In a meta-analysis, Lee et al. [5] showed that the patella height decreased significantly after HTO in a total of 831 OWHTO patients. Patellar infera leads to an increase in patellofemoral contact pressure, anterior knee pain, and patellar locking and a decreased range of motion. These changes eventually lead to the progression of patellofemoral joint OA [3, 16, 17]. Kim et al. [8] reported the prevalence of the progression of patellofemoral OA to be 41.2% in the femoral trochlea and 21.9% in the patella articular surface after more than 2 years in 114 patients who underwent OWHTO.

In many studies, the correction angle of the weight-bearing line in OWHTO is considered to be related to the change in patella height and the deterioration of the patellofemoral joint. Otsuki et al. [4] found that for every 1° increase in the correction angle, the CDI decreased by 1.7%, and CDI < 0.8 was a risk factor for the occurrence of a low patella after surgery. Yoon et al. [2] conducted a cohort study of 135 patients who had undergone OWHTO with an average of 52 months of follow-up. The study showed that the patella height decreased significantly after surgery, and patellofemoral joint OA progressed significantly more in the overcorrection group than in the undercorrection group and acceptable correction group. The authors suggested that overcorrection accelerated the decrease of the patella height and accentuated the patella tilt throughout the knee flexion process. Both patellar infera and patella tilt result in an increased contact pressure and contribute to the deterioration of the patellofemoral joint. We did not group the patients by the degree of correction due to the small number of patients, but the results showed that the corrected WBLR (at an average of 58.77%) did not affect patellar height or patellofemoral joint congruity, and the cartilage lesions of the patellofemoral joint did not tend to deteriorate. To prevent an increase in the lateral contact pressure of the patellofemoral joint, some studies have suggested that performing lateral retinacular release before knee osteotomy is helpful [18, 19]. Lateral retinacular release can be indicated on the basis of clinical symptoms and radiological manifestations, and it can improve patella alignment and relieve pressure on the patellofemoral joint. However, patients who had undergone lateral retinacular release were not included in the study because the change in the patellar position caused by the release would affect the accuracy of the data and results.

To prevent patellar infera, some studies [10, 11, 20, 21] have suggested that modified biplanar OWHTO is performed. In cases in which a separate descending osteotomy is performed by leaving the tuberosity attached to the proximal tibia, the patella height is not influenced, preventing the progression of patellofemoral joint OA. Additionally, during DTT-HTO, which was performed in this study, the tuberosity can remain attached to the proximal tibia to prevent patellar infera when the osteotomy region is located at the distal tibial tuberosity. DTT-HTO is simpler than biplanar OWHTO, and it is more convenient to adjust the weight-bearing line during this surgery, leading to a larger range of correction. However, the single plane of the osteotomy region in DTT-HTO involves a wide area of cortical bone. Therefore, additional fixation is needed to provide a sufficient anti-rotation force, which may increase the risk for lateral cortical fractures and slower bone healing. With the development of surgical techniques and internal fixation in recent years, π-plates and holes drilled along the cortical bone have been used in DTT-HTO, and the disadvantages of this surgery have gradually been overcome.

Although many studies have reported the effect of HTO on patella height, the correlations between different surgical procedures and the change in patella height are still controversial, and some researchers believe that the differences in the correlations are related to the differences in methods used to measure patella height, as there is no standard [22, 23]. The main methods used to measure patellar height are the Blackburne-Peel index (BPI), Insall-Salvati index (ISI), and Caton-Deschamps index (CDI). Because of the effect of HTO on the tibial plateau, the position of the tibial tubercle, and the patellar tendon adherence, it is necessary to consider whether the measurement can adequately represent the patella height. Some studies have suggested that when patellar height is measured, the tibial plateau plane rather than the patellar tendon should be taken as the reference [24]. BPI is difficult to measure accurately, resulting in scarring down or contractures of the patellar tendon, and the insertion point of the distal patellar tendon is difficult to identify by X-ray. The measurement of ISI has been shown to be affected by changes in tibial slope [25]. Therefore, the CDI was the measurement index selected for this study. The patellar height data are relatively stable and reproducible before and after surgery.

Our study has several limitations. (1) The level of evidence in retrospective studies is inadequate, and the mean follow-up time was only 23 months, which was not conducive to radiological and clinical evaluations of osteoarthritis degeneration. (2) Osteoarthritis is a degenerative disease that naturally progresses, and the incidence rate in females is significantly higher than that in males in China, so the duration of follow-up and sex of the patients included in studies may affect the results. (3) Although patients with lateral retinacular release were excluded from the study, changes in the internal environment of the knee joint caused by arthroscopy treatment may still have had an impact on patellofemoral joint congruity and patellofemoral joint OA. (4) Long-term follow-up studies with large sample sizes are needed to confirm whether the degree of correction in postoperative alignment after DTT-HTO affects patellar height and patellofemoral joint congruity.

## Conclusion

DTT-HTO can effectively prevent patellar infera, and its effects on postoperative patellofemoral joint congruity and patellofemoral joint OA progression are inconspicuous. It can be recommended as a treatment of varus knee combined with patellar infera or patellofemoral joint OA.

## Data Availability

The data and materials used and/or analyzed during the current study are not publicly available but available from the corresponding author on reasonable request.
